# Corneal topography in keratoconus: state of the art

**DOI:** 10.1186/s40662-016-0036-8

**Published:** 2016-02-22

**Authors:** F. Cavas-Martínez, E. De la Cruz Sánchez, J. Nieto Martínez, F. J. Fernández Cañavate, D. G. Fernández-Pacheco

**Affiliations:** Department of Graphical Expression, Technical University of Cartagena, C/Doctor Fleming s/n, Cartagena, 30202, Murcia Spain; Faculty of Sports Science, C/ Santa Alicia s/n, Santiago de la Ribera, 30720 Murcia, Spain

**Keywords:** Placido disc, Detection system, Topographer, Diagnosis

## Abstract

The morphological characterization of the cornea using corneal topographers is a widespread clinical practice that is essential for the diagnosis of keratoconus. The current state of this non-invasive exploratory technique has evolved with the possibility of achieving a great number of measuring points of both anterior and posterior corneal surfaces, which is possible due to the development of new and advanced measurement devices. All these data are later used to extract a series of topographic valuation indices that permit to offer the most exact and reliable clinical diagnosis. This paper describes the technologies in which current corneal topographers are based on, being possible to define the main morphological characteristics that the keratoconus pathology produces on corneal surface. Finally, the main valuation indices, which are provided by the corneal topographers and used for the diagnosis of keratoconus, are also defined.

## Background

Despite earlier works in the study of the human cornea, the first morphological characterizations for clinical diagnosis were made through the simple observation of the external appearance of the ocular surface [1]. At the beginning of the XVII century, Schiener [2] proposed the first device for the characterization of corneal morphology, comparing the reflection of the image generated by the corneal anterior shape with different calibrated spheres. Later, in the mid-nineteenth century, the Goode keratoscope appeared and enabled a corneal examination using the lateral reflection of a bright square object projected onto the patient’s cornea. Current corneal topography is based on the development of the Goode keratoscope by Antonio Placido da Costa [3], who developed it in 1880. This device was a variant that allowed a precise measurement of the anterior corneal surface through the projection of a circular plate onto the cornea. This plate, supported by a handle, contains a series of concentric rings and a hole in its center, allowing the doctor to see the reflected catoptrics image of the corneal surface of a patient located back to the light incidence. In the late nineteenth century, Javal performed the first qualitative analysis of the corneal morphology, joining Placido discs to his ophthalmometer and installing a viewer to obtain a wider field of the corneal image; he also proposed to photograph the image generated and to represent it on a diagram, which was aimed to analyze the variations in the curvature observed in the corneal surface. In 1896, Gullstrand [4, 5] presented the first quantitative assessment of the Placido discs’ images. He joined the discs to his photokeratoscope and examined the corneal photographs using a microscope in order to calculate the corneal curvature by a numerical algorithm.

In the early 80s, images were digitalized by hand and later computationally analyzed. Then, with the development of new computer equipment, the automation of this process appeared by capturing the image with a digital camera and its immediate computational analysis. The first fully automated corneal topographer was called Corneal Modeling System (CMS-1), developed by Computed Anatomy Inc. (New York, USA).

This article discusses the current technologies of corneal topographers and is aimed at: i) describing how they enable the main morphological measurements that produce the pathology of keratoconus on the corneal surface to be performed and ii) enumerating the main assessment indices provided by the topographers and used to diagnose keratoconus.

## Review

### Corneal topography: current technologies

Corneal topography is classically defined as a non-invasive exploratory technique to analyze both qualitatively and quantitatively the morphology of the cornea [6], enabling its geometric characterization and differentiating standard patterns from those potentially devastating for vision disorders caused by pathological ectatic conditions [7–11]. Current corneal topographers are based on one of these technologies: i) systems based on the light reflection on the cornea, ii) systems based on the projection of a slit light onto the cornea, and iii) systems based on the asymmetric reflexion of multicolor light-emitting diodes (LEDs).

#### Systems based on the light reflection on the cornea

Corneal topographers based on this technology, also called videokeratoscopes, are based on the application of the principles of convex mirrors’ geometrical optics to an instrument in which the rings or Placido discs with known size and spacing are reflected on the anterior surface of the cornea. This image is initially captured by a digital camera and then processed by a computer. From identifying the edges of the rings, each topographer uses an algorithm reconstruction of the corneal curvature, which accuracy depends on how the programming architecture is defined. The so-called arc-step algorithms are the most used and are based on an iterative process that uses a sequence of arcs from point to point, covering the entire corneal region from the apex to the periphery. This process does not ignore data obtained in the previous step (step i-1) for obtaining current data (step i) [12].

**Fig. 1:**
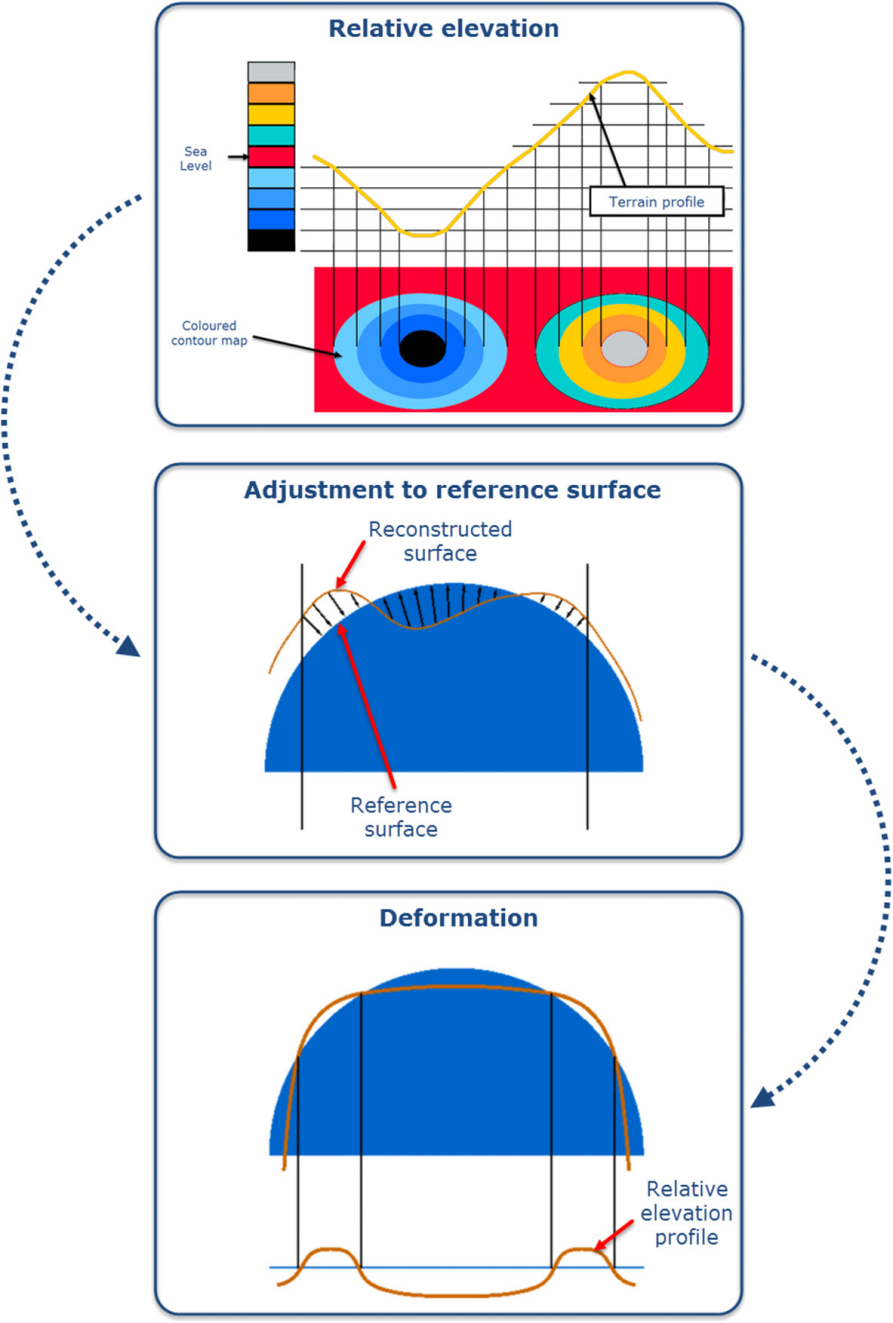
Corneal topography

Height and slope data derived from the radial curvature of the corneal surface are presented by topographers as corneal keratometric data of the entire surface by a series of maps that follow a color scale developed by the University of Louisiana [13] (Fig. 1).

- Cool colors correspond to flat curves and elevation values below the reference sphere (blue or violet colors).
- Mild colors correspond to medium curvature and elevation values equal to the reference sphere (green or yellow colors).
- Warm colors correspond to high curvature and elevation values above the reference sphere.

In addition, depending on the size of the Placido disc rings there are two commercial options [14]:

- Corneal topographer based on large diameter Placido disc rings. These devices are less susceptible to error associated with the misalignment between examiner and patient because they work at a large distance from the eye. However, because of this distance, it is possible to lose representative points because of the patient’s facial morphology, produced by the shadow of the patient’s nose and eyelashes.
- Corneal topograper based on small diameter Placido disc rings. These devices are most susceptible to alignment errors between the examiner and patient because they work in short distances, very close to the human eye. However, it mitigates the loss of information produced by patient’s facial morphology, reducing the shadow that the nose and eyelashes could cause.

However, both systems have an important limitation that results from the use of internal algorithms that do not allow an accurate characterization of the corneal morphology in case of high levels of irregularity. In some cases, it is possible to obtain mistakes up to 4 diopters in corneas that present a very curved morphology, as it occurs in keratoconus disease [15].

#### Systems based on the projection of a slit light onto the cornea

**Fig. 2:**
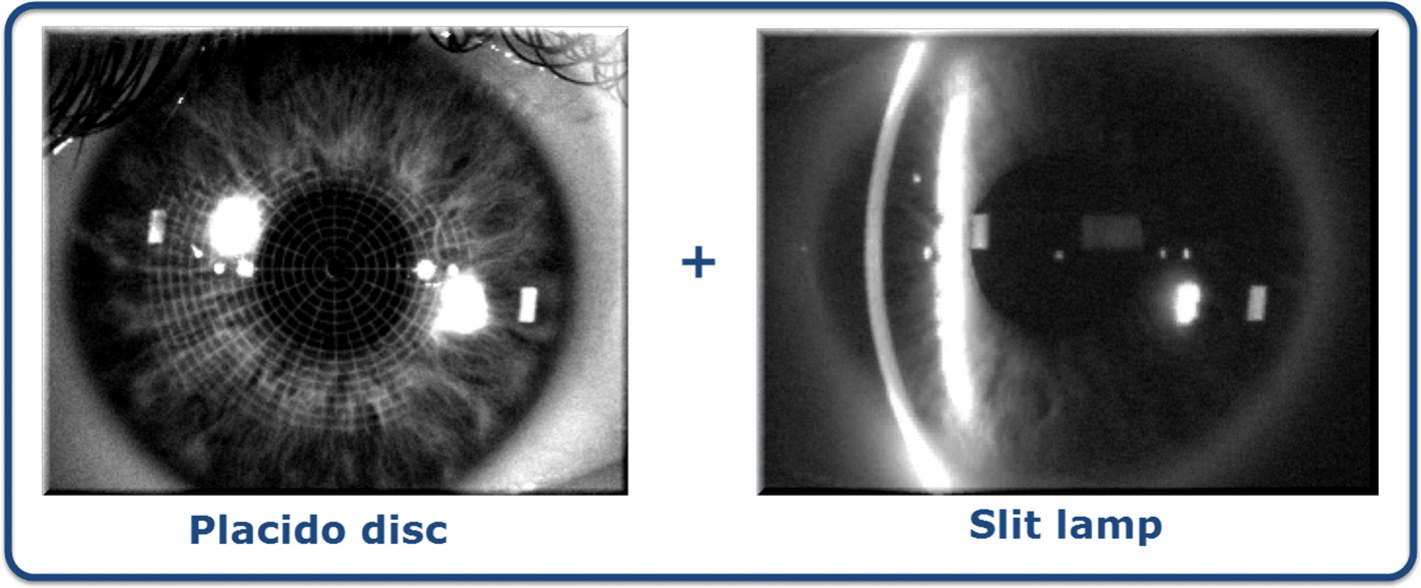
Systems based on the projection of a slit light on the cornea

These corneal topographers are based on the integration of a dual technology. The first process involves projecting a Placido disc, obtaining the mirror image by reflection and representing the curvature and refractive power, which is obtained by an arc-step algorithm. The second phase is the projection of a slit light onto the cornea. Due to the transparent structure of the cornea, and using Rayleigh scattering, it is possible to photograph it. These images will provide accurate data of corneal elevations for the entire anterior segment (see Fig. 2) [16].

**Fig. 3:**
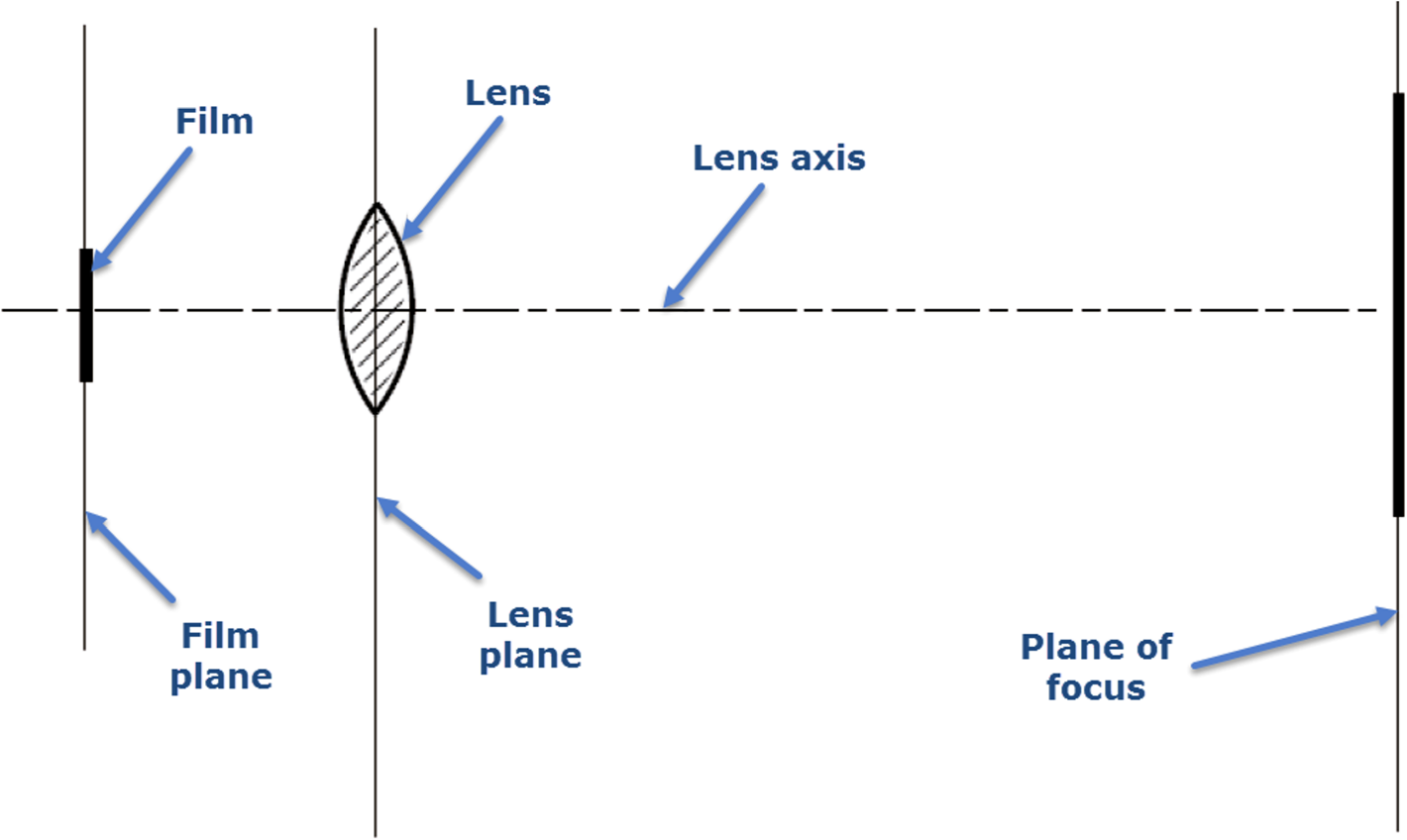
Principle of standard photography
- Systems based on the principle of Scheimpflug photography. Its main feature is that the plane of the camera lens is placed sideways to the image. The imaginary extensions of the planes of the film, the lens and the focal plane are not parallel (Fig. 4) [18], and therefore the focused region is increased and the image sharpness is improved [10, 21, 22]. The main commercial systems based on this principle are Pentacam (Oculus, USA), Galilei (Ziemer, Switzerland) and Sirius (CSO, Italy), which offer repeatable measurements of the corneal curvature and other anatomical measurements of the anterior segment. However, several authors question the degree of concordance between the measurements provided by these devices [23–25].

Furthermore, this technology has two variants, depending on the spatial arrangement of the photographic system:

- System based on the principle of standard or normal photography. Its main feature is that the plane of the camera lens is located in parallel with the image. It means that only a small region is focused (the imaginary extensions of the film planes, the lens and the focal plane are parallel) (Fig. 3) [17, 18]. The most common system is the Orbscan (Bausch & Lomb Incorporated, USA), which was the first commercial device that assessed the posterior corneal surface in a non-invasive and quick way. This system provides different maps of the anterior and posterior corneal surfaces, and also pachymetric data. However, several authors from scientific literature present a strong controversy due to the reliability of the measurements performed by this device on the posterior surface, and also the limited repeatability [13, 19, 20].

**Fig. 4:**
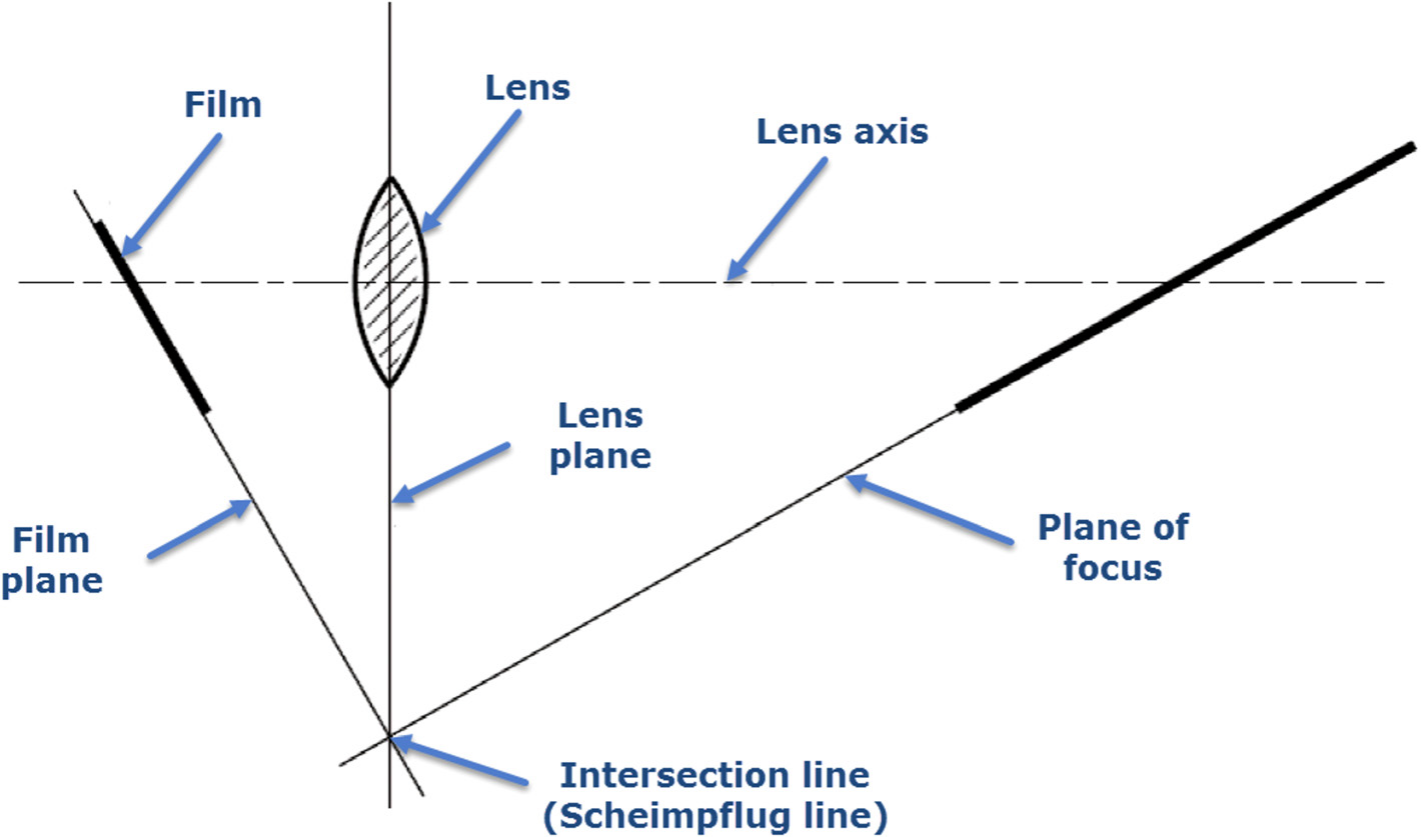
Principle of Scheimpflug photography

#### Systems based on the asymmetric reflexion of multicolor LEDs

These systems are based on the same optical principle than convex mirrors, but in this case the emitters are multicolor LEDs.

More specifically, this system uses a panel formed with an asymmetric distribution of more than 700 LEDs in colors red, yellow and green, which reflexion on the cornea provides a more accurate reconstruction of the corneal surface if compared with the lack of detail obtained when projecting a monochromatic light [26–28].

The main novelty of this system is the use of a unique reconstruction algorithm for the reflexion of each LED projection; this one being independent of the algorithm used for the other LED projections. This new approach has one main difference with respect to the systems previously described where the rest of them were based on the projection of a monochromatic light and use the same reconstruction algorithm for all the points of the corneal surface. Moreover, the new device permits redefining at the local level, every three points identified in the corneal surface, its curvature or elevation.

The unique commercial equipment based on this principle and currently available is the Cassini color LED Corneal Analyzer (i-Optics, The Netherlands) [26–28].

### Errors

The use of previously described corneal topography technologies involves the generation of errors during the acquisition process and data handling, and can be distinguished between intrinsic and extrinsic errors.

#### Intrinsic errors

These errors are derived from the equipment itself, so the measurements performed almost always include errors, as noise. This should be taken into consideration in data handling and data analysis [29–34]. Thus, devices show limitations derived from the measurement errors and digitalization [35], and also due to the internal algorithms making assumptions, rounding or approximation hypothesis. For this reason, it is necessary to consider that high levels of irregularity in corneal morphology, as a result of ectatic corneal pathology, could lead to a poor representation of the actual geometry of the cornea [15, 31, 34, 36]. In the case of insufficient data for the reconstruction of the corneal surface, these are internally provided by the device, which can lead to a biased characterization [36–38].

All these factors cause additional contamination of the measurement. In order to fix this problem, a pre-processing stage of the signal could be included, allowing the location and correction of the areas with high interference or incomplete data. But all the proposed solutions have a high computational cost, and are difficult to implement in real time, for instance, the use of bi-dimensional Gabor filtering to detect areas without directional pattern in the rings [39, 40] or the application of image processing techniques to improve data acquisition from areas with incomplete information [41].

Recent studies show that such errors can be reduced by new corneal topographers without a significant influence in data acquisition and processing [35, 42], so ophthalmologists accept the existence of these errors, which are considered negligible when we consider the clinical advantage that these topographers provide for the diagnosis of ectatic corneal diseases.

#### Extrinsic errors

These errors are derived from how the clinician or examiner handles the topographer, mainly during the approach and alignment of the system with the examined patient’s eye, and may lead to incorrect levels of astigmatism if wrongly performed. It can also fail due to an incorrect placement of the spatial center of the rings or the central crowns of Placido discs since curvature values are extremely sensitive to the misalignment or the fixation of the patient; thus, the accuracy of the measurements obtained by the device depends on good centering, alignment and focus by the clinician or examiner [43, 44].

Moreover, extrinsic errors derived from the quality of the tear film are also produced. Poor quality of the tear film together with the breakage in the distribution of the tear induce the appearance of anomalous data in the measures because of the distortion of the image of the Placido discs, which lead to the emergence of falsely irregular corneal spatial regions [45]. Also, alterations in the transparency of the cornea due to an ablation procedure as well as the existence of corneal scars or leucomas, may compromise the transparency of the cornea, resulting in the obtaining of erroneous pachymetric maps or altered curvatures maps, which may be confused with ectasia or irregular astigmatism [46].

Another source of error is the execution of the scanning process together with other clinical examination procedures. Recent research works have confirmed a significant variation in corneal parameters after instillation of different anesthetic eye drops. It has also been demonstrated that the dramatic reduction of the intrasession repeatability of measurements in this context, so this should be considered in order to avoid inappropriate clinical decision making [47].

Finally, another extrinsic error is derived from inadequate patient cooperation; a good eyelid opening during the examination is needed in order to obtain an adequate coverage of the corneal surface. In addition, the examiner should avoid the possible obstruction of the visual field resulting from patient’s eyelashes [48].

Current devices incorporate new technologies that provide an index to quantitatively assess the quality of each measurement based on the extrinsic errors described above. The measurement process will require repeats in those cases in which data does not meet a minimum standardized reliability criterion, or when it is not possible to do a correct interpretation of data for a proper diagnosis [43].

### Topographic analysis of keratoconus disease

Keratoconus is morphologically characterized on the anterior corneal surface by a cone-shaped protrusion [49, 50], generally eccentric with an inferior-temporal spatial orientation, which is physically interpreted as an area higher than the curve of the best adjustment surface in the elevation maps, and as an area more curved in the curvature map. This characteristic pattern of the corneal architecture is employed as a marker of severity of the keratoconus: keratometry values of 46 diopters or higher should be considered by the ophthalmologist as a sign of keratoconus disease [51]. The most frequent location of this corneal focal curvature is the mid-periphery region, both in the inferior-nasal and inferior-temporal quadrants. Specifically, the paracentral region comprises 72 % of cases, and approximately 25 % of those cases are included in the central region. Therefore, 97 % of keratoconus cases are located in the central and paracentral corneal regions, where presence of the protrusion in the peripheral region is being considered unusual [52–54].

The manifestation of this protrusion in the surface of the anterior shape generates a structural weakness in the cornea, which also implies a change in the morphology of the posterior corneal surface, showing an increased curvature when comparing with a healthy cornea, even during incipient stages [55]. There are several studies that have characterized and clinically evaluated the geometric correlation between anterior and posterior surfaces, both in healthy corneas [56, 57] and corneas with keratoconus disease [58]. Other studies have evaluated the curvature and the elevation of the posterior surface of the cornea in eyes with keratoconus to quantify this increase and assess whether these changes can be used as clinical tools for the diagnosis of subclinical keratoconus [55, 59].

Corneal topographers provide different maps to represent the measurements that characterize the surface of a keratoconic cornea. The ophthalmologist has to decide what scale should be considered in each case in order to get the best information from a clinical point of view [47]: the absolute scale gives the entire diopter range that the topographer can measure on a color scale, so it loses sensitivity to small changes whereas the relative scale adjusts the dioptric measurement range for each cornea, so it is sensitive to small changes and is more appropriate for a customized analysis of corneal morphology [7, 9, 52, 60].

**Fig. 5:**
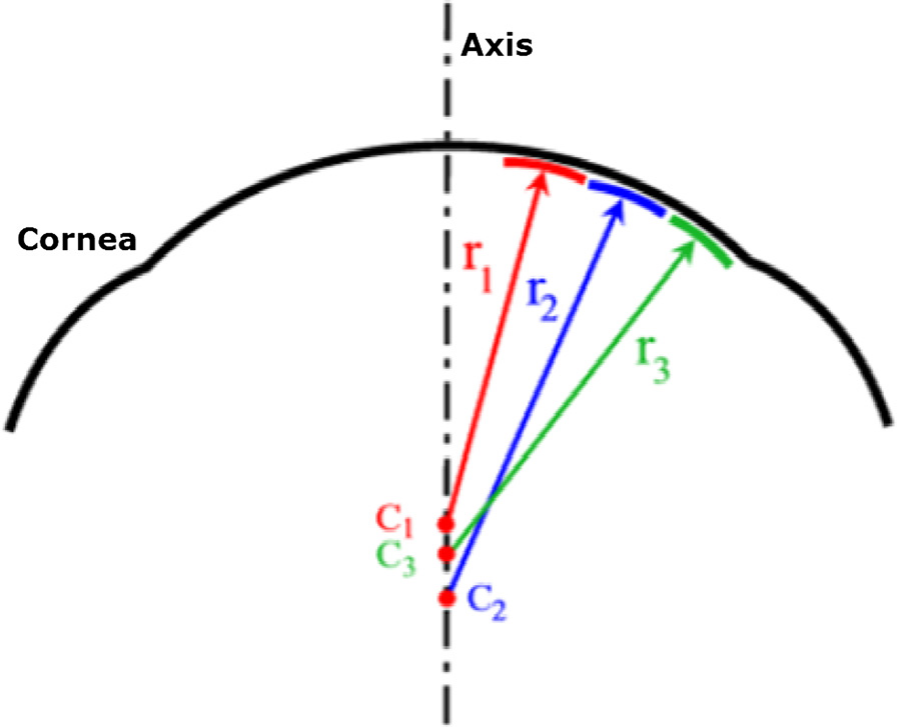
Geometric reconstruction of the corneal sagittal map
  - Tangential map. This map does not assume the spherical morphology of the cornea and therefore, the tangential curvature algorithm reconstructs the corneal surface by means of local curvature radii whose centers are not located on the optical axis. This map represents more accurately the curvature of the peripheral corneal region (Fig. 6) [47]. This map also shows a high sensitivity to data obtained, being suitable for monitoring the conical shape of the ectatic disease; however, it is less intuitive than the axial map and its interpretation may be more complex.

Current topographers provide, among others, the following maps:

- *Curvature keratometric maps*. They provide information about curvature at each point of the corneal surface, and can be axial (or sagittal) and tangential (or instant). Although both types report information about focal curvature, there exist significant differences between them [9, 61]:
  - Sagittal map. This type of map fixes the curvature centers on the optical axis and considers the corneal surface to have a spherical geometry, achieving a marked overall smoothing of the corneal periphery. This results in a larger and more peripheral curving area than the actual area of the cone. However, this consideration is erroneous and only true in the paraxial approach since the cornea has a spherical surface. Therefore, it distorts the true picture of the cornea and provides quantitatively inaccurate values [9, 47, 61, 62]. However, this map is useful for qualitative assessment through colors due to it softening the geometric contours of the cornea and facilitating the interpretation of the results by less-experienced users [47] (Fig. 5).

**Fig. 6:**
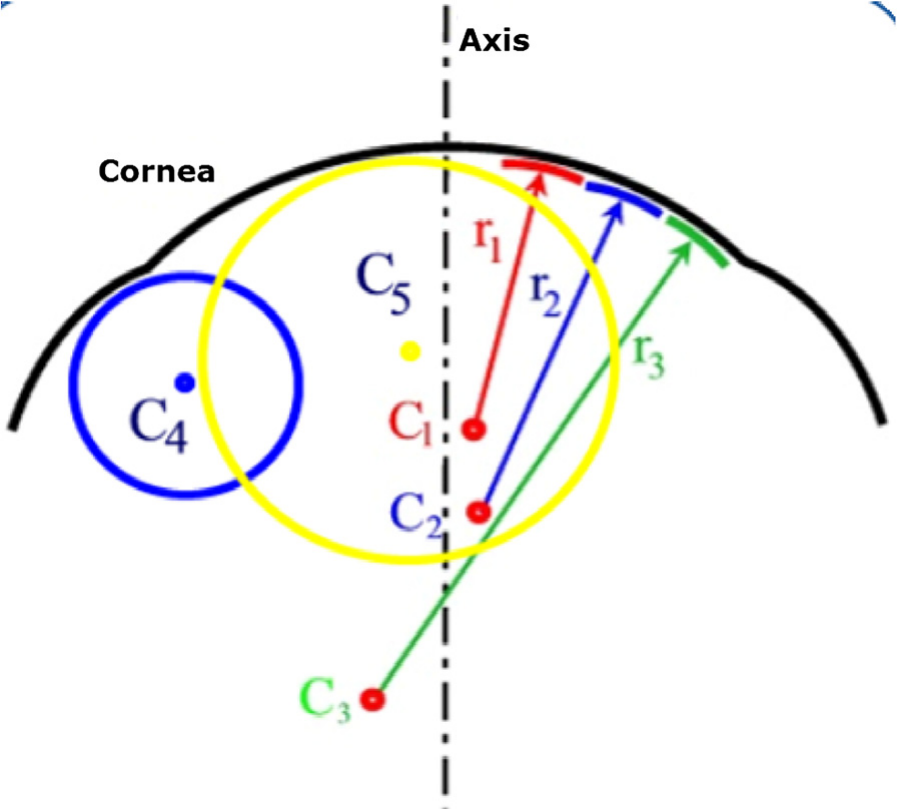
Geometric reconstruction of the corneal tangential map
- *Elevation maps*. These maps do not represent data directly measured by the corneal topographer, but are obtained by comparing the reconstruction of the anterior or posterior corneal surface to the best fitted surface, typically a sphere, a toroid, a revolution ellipsoid or a non-revolution ellipsoid. The difference between both surfaces is provided by altimetry data that correspond to the elevation maps (Fig. 7) [14, 63, 64]. In addition, typical dimensions of the reference surface are around 8 mm in diameter, so scenarios that may influence the data acquisition process, such as shadows generated by eyelashes, are avoided [48]. These maps present several advantages: i) data are presented quantitatively in μm, so they are highly accurate and have a high sensitivity to small changes that can occur in the corneal morphology as a result of keratoconus [9], ii) topographers allow the selection of the best suited surface to perform the elevation map, which results in a sensitivity increase of the clinical diagnosis. This map provides, for both corneal surfaces, the elevation of the corneal apex, the elevation of the minimum thickness point [64] and the elevation of the center of the central region [65]. As the posterior surface is not altered by the excimer laser photoablation or by the generation of the corneal flap, nor is adulterated by the hyperplastic effect of corneal epithelium, data from the posterior surface could be very useful for the clinical diagnosis of keratoconus [9, 13, 47, 65, 66].

**Fig. 7:**
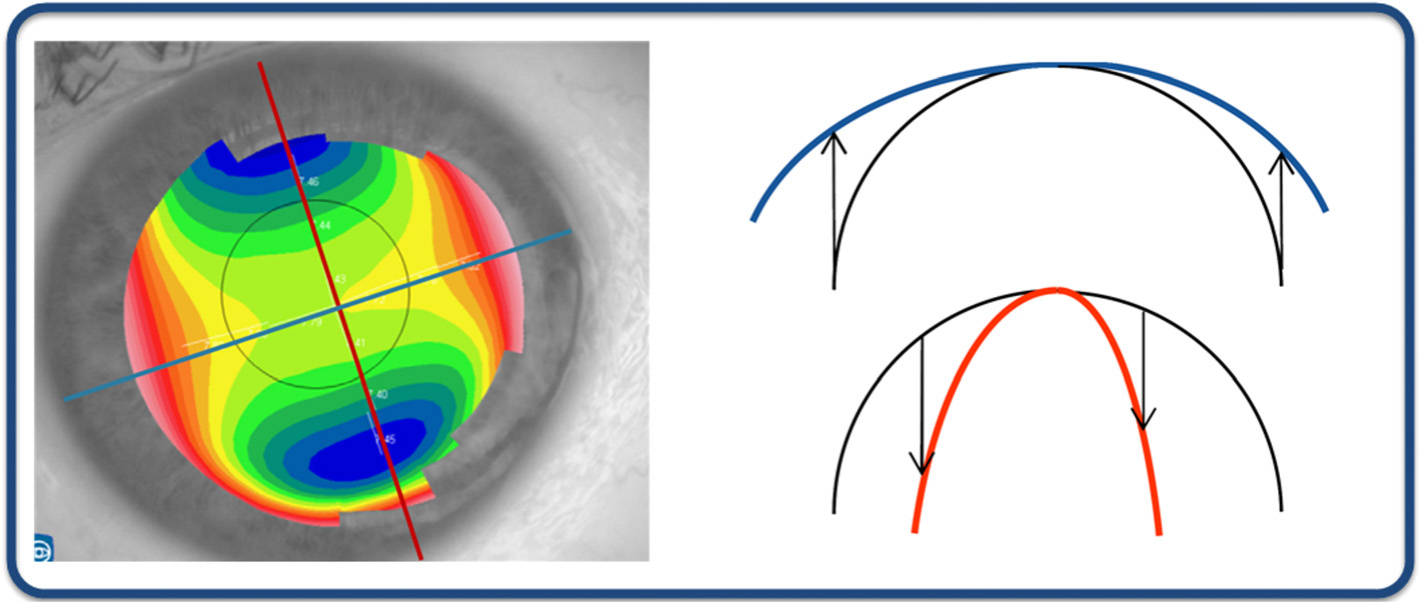
Altimetry elevation map
- *Thickness map*. This map does not show data directly measured by the corneal topographer, but must be precisely reconstructed using the anterior and posterior corneal surfaces. This map gives information of the minimum thickness point and its position on the center of the cornea. This point is essential for maintaining the corneal structure and determining the progressive thinning of the cornea when the severity of keratoconus progresses [67, 68]. Some studies in the scientific literature evaluate the pachymetric profile from the center to the periphery depending on the average thickness of the concentric rings separated by 0.1 mm and centered at the minimum thickness point. This profile presents a more abrupt change in a pathological stage than in a healthy scenario [61, 69, 70].

### Indices for assessing irregularities in the corneal surface

The main use of corneal topography is the generation of indices that allows quantification of the level of irregularity of the corneal morphology, at a local or general level. From these data, there are clinical studies that have tried to determine cut-off values to distinguish between normal and pathological corneas as well as to define several severity degrees of keratoconus. However, the main problem with these indices lies in the fact that each index has a high degree of specificity for the corneal topographer for which it has been developed, and cannot be directly extrapolated to other corneal topographers. In scientific literature, there are numerous indices used for the diagnosis of keratoconus, which are known as univariate or multivariate detection systems. Depending on the analysis approach, it could be a single index or a combination of indices that allows the interpretation of the main topographic characteristics of the keratoconus disease.

#### Univariate quantitative detection systems

- *Simulated keratometry (SIMK).* This index provides information on the diopter power of the flattest and most curved meridians in the so-called useful region of the topographer (ring diameters between 3 and 9 mm). Numerically, it is expressed as K1 and K2, and the difference between the two values provides a quantitative value of corneal astigmatism. A clinical study provided SIMK mean values of 43.53 ± 1.02 D for a group of normal corneas, and established a cut-off value for keratoconus group twice the value of the standard deviation of the control group [14, 71].
- *Surface asymmetry index (SAI).* It is an index that indicates an average value of the power differences between the points spatially located at 180° from 128 equidistant meridians. A radially symmetrical surface has a value of zero, and this value increases as the degree of asymmetry is greater [14, 72, 73].
- *Surface Regularity index (SRI).* It is a local descriptor of regularity in a central area of 4.5 mm of diameter (it comprises the central ten rings of Placido Disc). SRI quantifies power gradient differences between successive pairs of rings in 256 equidistant semi-meridians. It correlates well with the value of visual acuity (*p* = 0.80, *P* <0.001), assuming the cornea as the only limiting factor for vision. A normal cornea presents SRI values below 0.56 (this value would be 0 in a perfectly regular cornea) [14, 52, 72].
- *Central Keratometry (K Central).* This is the average value of corneal power for the rings with diameters of 2, 3 and 4 mm. Values below 47.2 D are considered normal, while values between 47.2 and 48.7 D are considered probable keratoconus. Values above 48.7 D are clinical keratoconus [14, 74].
- *Predicted Corneal Acuity (PC Acuity).* It quantifies the optical quality in Snellen units (with a range of 20/10 to 20/200) in the central zone of the cornea with 3 mm diameter [72, 75].
- *Inferior-Superior Value (I-S).* This index is defined as the power difference between five points of the inferior hemisphere and five points of the superior hemisphere of the corneal region located at 3 mm from the corneal apex at spatial intervals of 30°. A positive value indicates higher inferior curvature while a negative value indicates higher superior curvature. I-S values between 1.4 and 1.8 D are defined as cut-off points for suspected keratoconus and I-S values higher than 1.8 D as cut-off points for clinical keratoconus [74, 76].
- *Average corneal power (ACP).* This index indicates an average power value of various points in the central corneal region [74].
- *Topographic irregularity (IT).* It is the root mean square (RMS) value of the difference between the actual topography data and a geometric reference surface, which in this case is a spherical cylinder that best fits the corneal surface of the study [72].
- *Skew of steepest radial axis (SRAX).* It measures the angle between the more curved superior semi-meridian and the more curved inferior semi-meridian. The more curved semi-meridian for each hemisphere is determined by averaging the powers of the rings from 5 to 16 mm diameter. The smallest angle between these semi-meridians is subtracted from 180°, and the result, in degrees, is the SRAX index. A value greater than 20° is considered indicative of keratoconus, but due to the high dispersion of values in some astigmatic corneas, this value is only valuable if corneal astigmatism is greater than 1.5 D [72].
- *Corneal uniformity index (CU).* It corresponds to an index that quantifies the distortion uniformity in the central area with 3 mm diameter. It is expressed as a percentage, that is, a value of 100 % indicates that the cornea has a perfect consistency [75].
- *Irregular astigmatism index (IAI).* It is a measure of dioptric variables along each semi-meridian, which is normalized by the mean power of the cornea and the number of measured points [72, 74].
- *Apex curvature (AK).* It corresponds to an index that provides a value of the instantaneous curvature in the corneal apex. Values below 48 D indicate normality, values between 48 and 50 D are suspect, and values above 50 D denote abnormally high curvature [72].
- *Asphericity coefficient (Q).* It is an index that describes how the corneal curvature changes from the central region to the peripheral region. Its value depends on the diameter of study, so it is necessary to indicate it when expressing a particular index [75]. The most common parameter used in literature is Q. In a natural or non-pathological stage, the section of the cornea is a prolate ellipse with an average Q value setting −0.20 ± 0.12 [77, 78]. This is physically interpreted as the cornea is more curved in the center than at the periphery. For keratoconic corneas, Savini et al. [24] reported Q values in the corneal region that comprise a 8 mm diameter of −0.84 for the anterior surface and −1.10 for the posterior surface. Another study comprising the same diameter and the same corneal region reports Q values of the anterior surface between −0.65 and −1.18 and between −1.17 and −0.66 for the posterior surface [79], according to the severity of keratoconus using the Amsler- Krumeich classification [80, 81]. The values of asphericity describing the geometry of the cornea showed that the central and paracentral regions are more curved than the peripheral region, and this difference is greater in keratoconus disease than in normal corneas. Moreover, these values are higher when the severity of keratoconus increases. However, due to asphericity is measured in the central region (4–5 mm), if protusion is located in the peripheral region, the topographer may provide normal or even positive values of asphericity. Therefore, this quantitative index is not very specific for the diagnosis of keratoconus, and must be considered in relation to the apex position in the cone.
- *Effective refractive power (EffRP).* Index averaging the power in the central area of 3 mm diameter. It considers Styles-Crawford effect [75].
- *Corneal Irregularity Measurement (CIM).* This is a numeric index representing the degree of irregularity in the morphology of the corneal surface. It quantifies the standard deviation between the corneal surface and the best-fit reference surface, which in this case is a toric surface. High values of this ratio indicate a greater possibility of the cornea to present a pathology related to a morphological abnormality. A healthy cornea has CIM values from 0.03 to 0.68 μm, while a value from 0.69 to 1 μm is considered as suspect or normal limit, and a value from 1.10 to 5.00 μm as pathological or unusual [14].
- *Analyzed area (AA).* It is the ratio of the data area interpolated by the area circumscribed by the outermost peripheral ring [72, 74].
- *Mean toric keratometry (MTK).* This index is obtained from the data of the elevation map of the cornea. It compares and analyzes the elevation values of the cornea calculated by means of the best adjustment to a toric reference surface. If the MTK value is high, the cornea acquires a geometrical behavior similar to a toric surface, which is clinically interpreted as a major probability of suffering ectatic alterations. The MTK index follows a Gaussian probability distribution, with an average value of 44.5 D, and the range defined for the values from 41.25 D to 47.25 D contains 96 % of the population. [82].
- *Centre Surround index (CSI).* It is an index that quantifies the average power difference between the central zone of 3 mm diameter and a half-peripheral ring that is 3 and 6 mm diameters [72, 74].
- *Different sector index (DSI).* It is an index that quantifies the average power difference between sectors of 45° with the highest and lowest power [72, 74].
- *Opposite industry index (OSI).* This is another index indicating in terms of average power difference between opposing sectors of 45° [72, 74].
- *Calossi-Foggi Apex curvature gradient (ACG).* This index quantifies the average difference per length unit of the corneal power in relation to the apical power. Values greater than 2 D/mm can indicate keratoconus, between 1.5 and 2 suspect, and less than 1.5 is considered a normal value [72].
- *Calossi-Foggi Top-Bottom Index.* It is a vertical asymmetry index similar to I-S value, but this indicates the difference in terms of average power between the superior area and an inferior area. This last is determined according to a major probability of presence of the cone. A positive value means that inferior area is more curved, and vice versa. A value less than 1.5 is considered normal, a value between 1.5 and 2 considered suspect, and values greater than 2 are considered abnormal [72].
- *Orbscan surface irregularity.* It is a coefficient calculated from the standard deviations of the mean curvature and mean astigmatism in the zones comprised between the center rings of 3 and 5 mm diameter. Values greater than 1.5 in the area of 3 mm and/or 2 in the area of 5 mm are indicative of high irregularity. This value is not specific for keratoconus, but merely indicates irregularity [72].

### Multivariate quantitative detection systems

The diagnosis of keratoconus has been facilitated by the application of different quantitative detection systems using multivariate combinations of the topographic indices described above. Each topographer incorporates a different strategy and it is essential for the user to understand the sensitivity and specificity of each software. These systems can be:

- *KISA%*. It is calculated from four indices: Central K, SIMK, I-S and SRAX. It is a very effective index in the identification of keratoconus, but may have a significant number of false negatives in the clinical diagnosis and in incipient keratoconus cases, which can suppose a major risk in its use as a screening tool in refractive surgery. A value between 60 and 100 is indicative of suspected keratoconus, while a value greater than 100 is diagnostic of keratoconus; for this last value the KISA index has a high sensitivity and specificity [14, 72, 83].
- *Chastang method*. Combines SDSD and Asph indices, developing a primary decision tree. This method is not applicable for defining the grade or severity of keratoconus, so incipient or early cases are not identified [72, 84].
- *Belin/Ambrósio Enhanced Ectasia Display III (BAD III)*. This method combines the following nine parameters: anterior elevation at the minimum thickness point, posterior elevation at the minimum thickness point, change in anterior elevation, change in posterior elevation, corneal thickness at minimum thickness point, location of thinnest point, pachymetric progression, Ambrósio relational thickness and Kmax. The BAD III index provides individual information for every parameter and then performs a discriminant analysis combined with a regression of the nine indices, which permits the discrimination between healthy and keratoconic corneas [85].
- *Keratoconus severity index (KSI)*. This index is also known in the Ophthalmology field as the Smolek-Klyce method [71]. This multivariate system calculates the severity of keratoconus, which possibly distinguishes between a healthy cornea, a suspected keratoconic cornea and a cornea with keratoconus. The algorithm used is based on a neural network with 10 topographic indices as inputs. A KSI value <15 % is considered normal, values between 15 and 30 % as suspected keratoconus, and above this value is considered subclinical keratoconus [14, 71, 72].
- *Keratoconus Index (KCI)*. This is a method commonly known in the Ophthalmology field as the Klyce-Maeda method [86]. This method can differentiate a healthy cornea from a keratoconic cornea, and also distinguish between keratoconus developed in the central or the peripheral regions.
- *Keratoconus prediction index (KPI)*. It is calculated by a combination of 8 topographic indices and uses a linear discriminant function. These indices are: Sim K1, K2 Sim, UPS, DSI, OSI, CSI, IAI and AA. A value greater than 0.23 is suggestive of keratoconus. In a validation group of a hundred eyes with different clinical conditions, this method showed a sensitivity of 68 % and a specificity of 99 %. The KPI multivariate method along with DSI, OSI, GSI and Sim K2 indices were implemented using an expert computational algorithm based on a binary decision tree [72].
- *PathFinder Corneal Analysis*. It is a system which uses three different indices (CIM, Q and MTK) for detecting the morphological alterations that corneal topographies present. In the case of a normal cornea, the three indicators give values within normal limits. In the case of molding due to contact lenses, there is also a corneal distortion known as pseudo-keratoconus, where the CIM index is outside the normal range, but the other two indicators are within normal limits. In the case of a subclinical keratoconus, central curvature values and inferior-superior asymmetry cause both the CIM and the MTK to have abnormal values, while asphericity remains normal or borderline normal. In the case of keratoconus, the three indicators are outside the bounds of normality [14, 87].
- *Rabinowitz and McDonell Index*. It is one of the first multivariate indices based on the combination of the numerical values supplied by the I-S index, the value of the center, K and the difference of the central K between both eyes of the patient. This multivariate system combines the information obtained from the central curvature values with the inferior-superior asymmetry values as diagnostic parameters of keratoconus that can occur in both central and peripheral regions. However, this indicator is unable to quantify the amount of irregular astigmatism associated with the keratoconus disease [72, 76].

## Conclusions

One important characteristic of the corneal topography is the possibility of generating a series of indices that quantify the level of irregularity of the corneal morphology at the local or global level. Some previous clinic studies have used these data to calculate the cut-off values that permit the discrimination between normal and keratoconic corneas as well as to define several stages or grades of keratoconus. However, these indices present two problems. On one hand, each index has a high grade of specificity for the corneal topographer for which it has been developed, not being possible to extrapolate it directly to other corneal topographers. On the other hand, univariate or multivariate indices do not provide clinical parameters for the diagnosis of keratoconus with 100 % sensitivity and specificity. For that reason, and after reviewing the existing technologies and topographic indices described in the literature, the present paper proposes a new concept for the integral morphological analysis of the cornea in which several topographic indices are analyzed simultaneously with the aim of providing the most exact and reliable clinical diagnosis.
